# High serum cortisol level is associated with increased risk of delirium after coronary artery bypass graft surgery: a prospective cohort study

**DOI:** 10.1186/cc9393

**Published:** 2010-12-30

**Authors:** Dong-Liang Mu, Dong-Xin Wang, Li-Huan Li, Guo-Jin Shan, Jun Li, Qin-Jun Yu, Chun-Xia Shi

**Affiliations:** 1 Department of Anesthesiology and Surgical Intensive Care, Peking University First Hospital, No. 8 Xishiku Street, Beijing 100034, PR China; 2 Department of Anesthesiology, Cardiovascular Institute and Fuwai Hospital, Chinese Academy of Medical Sciences and Peking Union Medical College, No. 167 Beilishi Road, Beijing 100037, PR China

## Abstract

**Introduction:**

The pathophysiology of postoperative delirium remains poorly understood. The purpose of this study was to examine the relationship between serum cortisol level and occurrence of early postoperative delirium in patients undergoing coronary artery bypass graft (CABG) surgery.

**Methods:**

A total of 243 patients undergoing elective CABG surgery were enrolled. Patients were examined twice daily during the first five postoperative days and postoperative delirium was diagnosed by using the Confusion Assessment Method for the Intensive Care Unit (CAM-ICU). Blood samples were obtained between 7 a.m. and 8 a.m. on the first postoperative day and serum cortisol concentrations were then measured. Multivariate logistic regression analyses were performed to identify risk factors of postoperative delirium.

**Results:**

Postoperative delirium occurred in 50.6% (123 of 243) of patients. High serum cortisol level was significantly associated with increased risk of postoperative delirium (OR 3.091, 95% CI 1.763-5.418, *P *< 0.001). Other independent risk factors of postoperative delirium included increasing age (OR 1.111, 95% CI 1.065-1.159, *P *< 0.001), history of diabetes mellitus (OR 1.905, 95% CI 1.001-3.622, *P *= 0.049), prolonged duration of surgery (OR 1.360, 95% CI 1.010-1.831, *P *= 0.043), and occurrence of complications within the first day after surgery (OR 2.485, 95% CI 1.184-5.214, *P *= 0.016). Patients who developed postoperative delirium had a higher incidence of postoperative complications and a prolonged duration of postoperative ICU and hospital stay.

**Conclusions:**

Delirium was a common complication after CABG surgery. High serum cortisol level was associated with increased risk of postoperative delirium. Patients who developed delirium had outcomes worse than those who did not.

## Introduction

Delirium is a common complication after cardiac surgery. According to the *Diagnostic and Statistical Manual of Mental Disorders, Fourth Edition *(DSM-IV), delirium is a transient mental syndrome characterized by (a) disturbance of consciousness with a reduced ability to focus, sustain, or shift attention; (b) change in cognition (such as memory deficit, disorientation, or language disturbance) or development of a perceptual disturbance that is not better accounted for by a preexisting, established, or evolving dementia; and (c) disturbance developing over a short period of time (usually hours to days) and tending to fluctuate during the course of the day [1]. The reported incidences of delirium after cardiac surgery varied from 3% to 72% [2-5], and the occurrence of postoperative delirium is associated with multiple adverse effects, such as self-extubation, prolonged hospital stay, increased health-care costs, and high mortality rate [6-11].

Despite the numerous studies, the pathophysiology of delirium remains poorly understood [12,13]. As a universal phenomenon, delirium is frequently identified after major complicated surgery (including cardiac and vascular surgery) but rarely noted after minor ambulatory surgery (such as cataract surgery) [5,14-16]. These findings suggest that the stress response induced by surgical stimuli might play an important role in the pathogenesis of postoperative delirium.

Cortisol is one of the most important stress hormones in humans. Its secretion is proportional and positively correlated to the severity of surgical stimuli [17,18]. A reciprocal control, the hypothalamic-pituitary-adrenal axis, exists between the brain and glucocorticoid hormones. Under stressful conditions, the brain promotes adrenocortical function via hypothalamic corticotrophin-releasing hormone. On the other hand, glucocorticoids act at specific receptors in the hypothalamus, thus producing negative feedback mechanisms.

It has long been observed that high levels of circulating glucocorticoids might have harmful effects on the brain and cause psychiatric symptoms [19-21]. This is because there are glucocorticoid receptors in the hippocampus and frontal lobe, the regions that are closely associated with cognition. The effects of glucocorticoids on cognition follow an inverted U-shape dose response relationship; that is, memory is impaired by sustained glucocorticoid levels that are too low or too high but is improved by proportionate glucocorticoid level [22].

However, the relationship between circulating cortisol level and the occurrence of postoperative delirium has not been fully demonstrated. A recent study by Shi and colleagues [23] found that elevated serum cortisol level was associated with increased incidence of postoperative delirium in critically ill patients after noncardiac surgery. We suppose that the occurrence of postoperative delirium in patients undergoing cardiac surgery is also related to stress response and, thus, the elevated circulating cortisol level. The purpose of this study was to examine the association between serum cortisol level and occurrence of early postoperative delirium in patients undergoing coronary artery bypass graft (CABG) surgery.

## Materials and methods

The study protocol was approved by the clinical research ethics committees of Peking University First Hospital and Beijing Fuwai Hospital. All patients gave written informed consent.

### Patients

This was a prospective cohort study. The inclusion criteria were consecutive adult patients who were referred to Beijing Fuwai Hospital for elective CABG surgery from March 2008 to July 2008. The exclusion criteria were as follows: (a) previous cardiothoracic surgery, (b) history of schizophrenia, (c) history of adrenal gland disease, (d) history of glucocorticoid therapy for more than 7 consecutive days within 1 year, (e) preoperative left ventricular ejection fraction of less than 25% (echocardiography, Simpson's method), and (f) concomitant surgery other than CABG, such as valvular replacement.

### Anesthesia, surgery, and postoperative care

Patients were premedicated with midazolam (7.5 mg by mouth) and morphine (10 mg intramuscularly). Anesthesia was induced with fentanyl (5 to 10 μg/kg), etomidate (0.2 to 0.3 mg/kg), and rocuronium (0.6 mg/kg). Anesthesia was maintained with midazolam (0.1 to 0.2 mg/kg), fentanyl (20 to 30 μg/kg), isoflurane (0.5% to 1%), and propofol (2.4 to 4.0 mg/kg per hour during cardiopulmonary bypass). Muscle relaxation was maintained with supplemental doses of vencuronium. Intraoperative monitoring included 5-lead electrocardiogram, radial arterial pressure, central venous pressure, pulse oxygen saturation, end-tidal expiratory carbon dioxide, nasopharyngeal temperature, bladder temperature, and urine output. A pulmonary artery catheter was inserted when necessary.

The type of surgery (on-pump or off-pump surgery) and the number of bypass grafts were determined by the surgeons. All patients underwent CABG surgery through a median sternotomy. Aortic palpation was used to detect atherosclerosis and, if present, to select an appropriate site for cannulation and clamping. For patients undergoing off-pump surgery, distal anastomoses were performed with the help of an Octopus tissue stabilizer (Medtronic, Inc., Minneapolis, MN, USA). Proximal anastomoses were then fashioned onto the aorta by means of a single side-clamp. Nasopharyngeal temperature was maintained above 35°C, and systolic blood pressure was kept at 80 mm Hg or greater throughout the procedure.

For patients undergoing on-pump surgery, cardiopulmonary bypass was established with a Stöckert S3 roller pump (Stöckert Instrumente GmbH, Munich, Germany), a membrane oxygenator (Maxima Forte; Medtronic, Inc.), and a 40-μm arterial blood filter (Dideco, Mirandola, Italy). Moderate hypothermia (32°C) and α-stat acid-base management were used. Perfusion pressure was kept at 60 to 80 mm Hg, and a pump flow was maintained between 2 to 2.4 L/min per m^2^. After all distal anastomoses were completed, the aortic cross-clamp was removed, and proximal anastomoses were then performed by means of a single side-clamp on the aorta.

After surgery, all patients were transferred to the intensive care unit (ICU) intubated and were placed on mechanical ventilation. Propofol and morphine were routinely administered for sedation and analgesia. Midazolam was administered as required. Extubation and ICU discharge were decided by attending intensivists. Hospital discharge was decided by the attending surgeon. Patients were followed up until 28 days after surgery. Definitions of postoperative complications are shown in Table 1.

**Table 1:** Definitions of postoperative complications

Complications	Requirements for acceptance
Cardiac insufficiency	Requirement of inotropic support for more than 24 hours or intra-aortic balloon pump support or both
Arrhythmia	New-onset arrhythmia confirmed by 12-lead electrocardiogram and necessitated medical treatment or electroversion or both
Myocardial infarction	Increase of troponin T concentration above the hospital laboratory's myocardial infarction threshold and either new Q waves (duration of at least 0.03 seconds) or persistent changes (4 days) in ST-T segment
Respiratory insufficiency	Requirement of mechanical ventilation for more than 24 hours
Stroke	Appearance of persisted new focal neurologic deficit and confirmed by neurologic imaging study
Sepsis	Two or more of systemic inflammatory response syndrome criteria, with known or suspected evidence of infection
Pleural effusion	Confirmed by chest x-ray film and necessitated aspiration or surgical drainage
Surgical bleeding	Requirement of reoperation to stop bleeding

### Measurement of serum cortisol level

Blood samples were obtained between 7 and 8 a.m. on the first postoperative day. BD tubes (Becton, Dickinson and Company, Franklin Lakes, NJ, USA) were used throughout the study. Prior to the assay, the samples were kept refrigerated for no longer than 12 hours at 4°C. Serum cortisol concentration was measured with a solid-phase, competitive chemiluminescent enzyme immunoassay in a calibrated IMMULITE 1000 analyzer (Diagnostic Products Corporation, Los Angeles, CA, USA). The intra-assay and interassay coefficients of variation at various concentrations were less than 5.6% and less than 8.2%, respectively. The normal range is 138 to 690 nmol/L in the laboratory where measurements were performed.

### Delirium assessment

Delirium assessment was performed in two steps. First, level of sedation (level of arousal) was assessed by means of the Richmond Agitation Sedation Scale (RASS) [24,25]. This is a 10-point scale with four levels of anxiety or agitation (+1 restless to +4 combative), one level representing an alert and calm state (0), and five levels of sedation (-5 = nonarousable to -1 = drowsy). If the patient was deeply sedated or was unarousable (-4 or -5 on the RASS), assessment was stopped and then was repeated later. If RASS was above -4 (-3 through +4), assessment was continued to the next step. Second, delirium was diagnosed by means of the Confusion Assessment Method for the Intensive Care Unit (CAM-ICU) [26,27]. This is an instrument designed to diagnose delirium in nonverbal, critically ill patients. It indicates four features of delirium: (a) acute onset of mental status changes or a fluctuating course, (b) inattention, (c) disorganized thinking, and (d) altered level of consciousness. To meet the diagnostic definition of delirium, a patient must display both (a) and (b) and either (c) or (d).

Prior to the study, the physician performing the assessment of delirium (D-LM) was trained by a psychiatrist to use CAM-ICU. Definition and examples of delirium features were explained and discussed. For the purpose of training and standardization, eligible patients were randomly selected and each patient was independently evaluated by the investigator (according to CAM-ICU) and the psychiatrist (according to DSM-IV) during the same observational period. The process continued until agreement for the diagnosis of delirium reached 100%. During the study phase, patients were assessed for delirium twice daily (from 6 to 8 a.m. and from 6 to 8 p.m.). For each patient, delirium assessment was performed until the fifth postoperative day or the disappearance of delirious symptoms for two consecutive days. Study personnel who assessed delirium were blinded to the treatment details and the serum cortisol results.

### Statistical analysis

Continuous variables are presented as mean ± standard deviation (SD) or median (interquartile range). Data were compared with the use of the independent samples *t *test or the Mann-Whitney *U *test. Categorical variables are presented as number of patients (percentage). Data were compared with the use of the chi-square test or the Fisher exact test. The effect of serum cortisol level on the occurrence of postoperative delirium was assessed with the use of multivariate logistic regression analyses. Initially, baseline and perioperative variables were evaluated for univariate association with postoperative delirium. Variables that were significant in univariate analyses (*P *< 0.10) were included in a multivariate logistic regression model to determine the risk-adjusted predictors of delirium. Two-sided *P *values of less than 0.05 were regarded as significant. All statistical analyses were performed with the SPSS statistical package, version 14.0 (SPSS Inc., Chicago, IL, USA).

## Results

**Figure 1:**
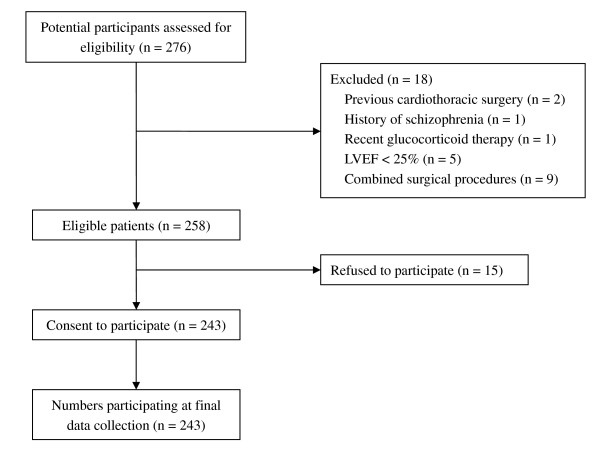
**Flow diagram of the study**. LVEF, left ventricular ejection fraction.

Two hundred seventy-six patients underwent elective CABG surgery during the study period, and 258 matched the criteria of selection. Among the eligible patients, 243 gave written consent and were enrolled in this study (Figure 1). The perioperative variables of all enrolled patients are listed in Tables 2 and 3.

**Table 2:** Preoperative variables

Variable	All enrolled patients (*n *= 243)	Non-delirious patients (*n *= 120)	Delirious patients (*n *= 123)	*P *value
Age, years	61.0 ± 8.3	58.3 ± 8.0	63.6 ± 7.7	< 0.001
Body mass index, kg/m^2^	26.1 ± 3.5	26.2 ± 3.9	26.0 ± 3.0	0.643
Education, years	10.4 ± 4.4	10.4 ± 4.3	10.4 ± 4.4	0.992
Female gender	43 (17.7%)	21 (17.5%)	22 (17.9%)	0.937
Previous medical history				
Hypertension	161 (66.3%)	75 (62.5%)	86 (69.9%)	0.221
Previous myocardial infarction^a^	114 (46.9%)	55 (45.8%)	59 (48.0%)	0.739
Diabetes mellitus	89 (36.6%)	36 (30.0%)	53 (43.1%)	0.034
Hyperlipidemia	96 (39.5%)	47 (39.2%)	49 (39.8%)	0.915
Arrhythmia	58 (23.9%)	26 (21.7%)	32 (26.0%)	0.426
Stroke	31 (12.8%)	11 (9.2%)	20 (16.3%)	0.097
COPD	6 (2.5%)	1 (0.8%)	5 (4.1%)	0.213
Renal dysfunction^b^	9 (3.7%)	4 (3.3%)	5 (4.1%)	1.000
Chronic smoking^c^	79 (32.5%)	40 (33.3%)	39 (31.7%)	0.787
Alcoholism^d^	34 (14.0%)	21 (17.5%)	13 (10.6%)	0.119
Habitual benzodiazepine use	23 (9.5%)	12 (10.0%)	11 (8.9%)	0.778
Previous general anesthesia	17 (7.0%)	9 (7.5%)	8 (6.5%)	0.761
Preoperative LVEF, percentage^e^	58.0 ± 9.6	59.4 ± 8.4	56.7 ± 10.5	0.024
Preoperative CCS class				0.736
I	53 (21.8%)	24 (20.0%)	29 (23.6%)	
II	126 (51.9%)	63 (52.5%)	63 (51.2%)	
III	53 (21.8%)	26 (21.7%)	27 (22.0%)	
IV	11 (4.5%)	7 (5.8%)	4 (3.3%)	
Preoperative NYHA functional class				0.089
I	77 (31.7%)	45 (37.5%)	32 (26.0%)	
II	131 (53.9%)	62 (51.7%)	69 (56.1%)	
III	35 (14.4%)	13 (10.8%)	22 (17.9%)	
Preoperative EuroSCORE score	2.6 ± 2.1	2.0 ± 1.8	3.2 ± 2.1	< 0.001

Data are presented as mean ± standard deviation or number of patients (percentage). ^a^Myocardial infarction of more than 1 month before surgery. ^b^Serum creatinine of greater than 177 μmol/L. ^c^Smoking of more than 20 cigarettes per day within 1 month. ^d^Consumption of an equivalent of 150 mL of alcohol per week. ^e^Results of echocardiography (Simpson's method). CCS, Canadian Cardiovascular Association; COPD, chronic obstructive pulmonary disease; EuroSCORE, European System for Cardiac Operative Risk Evaluation; LVEF, left ventricular ejection fraction; NYHA, New York Heart Association.

**Table 3:** Perioperative variables

Variable	All enrolled patients (*n *= 243)	Non-delirious patients (*n *= 120)	Delirious patients (*n *= 123)	*P *value
Duration of anesthesia, hours	5.00 ± 1.19	4.81 ± 1.13	5.18 ± 1.22	0.017
Dosage of fentanyl, μg/kg	24.9 ± 6.1	24.6 ± 6.0	25.2 ± 6.1	0.386
Dosage of etomidate, mg/kg	0.23 ± 0.08	0.23 ± 0.08	0.23 ± 0.08	0.970
Use of anticholinergic drugs	97 (39.9%)	49 (40.8%)	48 (39.0%)	0.773
Duration of surgery, hours	4.00 ± 1.16	3.81 ± 1.13	4.18 ± 1.16	0.011
On-pump surgery	114 (46.9%)	56 (46.7%)	58 (47.2%)	0.939
Coronary artery bypass grafts, number	3.3 ± 0.8	3.2 ± 0.8	3.3 ± 0.7	0.365
Blood transfusion of at least 400 mL	9 (3.7%)	1 (0.8%)	8 (6.5%)	0.036
APACHE II score^a ^	6.43 ± 3.25	5.56 ± 3.03	7.28 ± 3.24	< 0.001
Serum cortisol concentration, nmol/L	549.9 ± 300.2	473.6 ± 305.4	625.6 ± 275.9	< 0.001
Serum cortisol level^b^				< 0.001
Level 1	20 (8.2%)	12 (10.0%)	8 (6.5%)	
Level 2	145 (59.7%)	90 (75.0%)	55 (44.7%)	
Level 3	78 (32.1%)	18 (15.0%)	60 (48.8%)	
Duration of sedation, hours	10.0 (7.0-13.0)	9.5 (7.0-12.0)	10.5 (7.8-14.0)	0.045
Use of benzodiazepines	63 (25.9%)	28 (23.3%)	35 (28.5%)	0.362
Duration of mechanical ventilation, hours	14.8 (12.2-18.0)	13.9 (11.9-15.8)	16.0 (13.2-19.3)	< 0.001
Postoperative LVEF, percentage^c^	54.8 ± 8.7	56.3 ± 8.5	53.4 ± 8.7	0.008
Postoperative complications				
Cardiac insufficiency	48 (19.8%)	16 (13.3%)	32 (26.0%)	0.013
Arrhythmia	18 (7.4%)	5 (4.2%)	13 (10.6%)	0.057
Myocardial infarction	1 (0.4%)	1 (0.8%)	0 (0.0%)	0.494
Respiratory insufficiency	4 (1.6%)	0 (0.0%)	4 (3.3%)	0.122
Surgical bleeding	5 (2.1%)	1 (0.8%)	4 (3.3%)	0.370
Sepsis	5 (2.1%)	0 (0.0%)	5 (4.1%)	0.060
Pleural effusion	13 (5.3%)	7 (5.8%)	6 (4.9%)	0.741
Stroke	1 (0.4%)	0 (0.0%)	1 (0.8%)	1.000
Postoperative complications within 1 day^d^	67 (27.6%)	21 (17.5%)	46 (37.4%)	0.001
Postoperative complications within 5 days^d^	82 (33.7%)	26 (21.7%)	56 (45.5%)	< 0.001
Postoperative complications within 28 days^d^	86 (35.4%)	28 (23.3%)	58 (47.2%)	< 0.001

Data are presented as mean ± standard deviation, number of patients (percentage), or median (interquartile range). ^a^Scored using worst values over the first 24 hours after surgery. ^b^The normal range is 138 to 690 nmol/L. Level 1 indicates a serum cortisol concentration of less than 138 nmol/L, level 2 between 138 and 690 nmol/L, and level 3 of greater than 690 nmol/L. ^c^Results of echocardiography (Simpson's method) performed before discharge from the hospital. ^d^Complications that occurred before or during the 1st, 5th, or 28th postoperative day. APACHE II, Acute Physiology and Chronic Health Evaluation II; LVEF, left ventricular ejection fraction.

**Figure 2:**
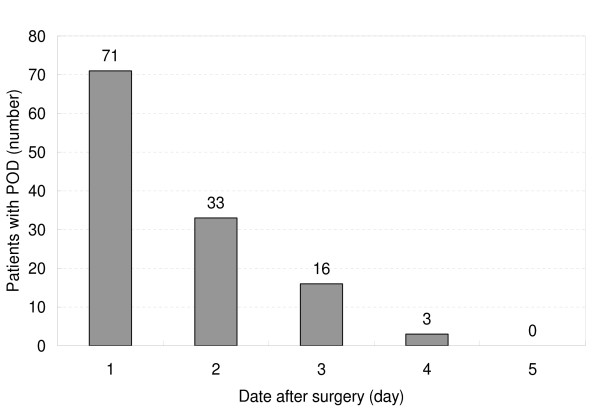
**Number of patients whose first episode of delirious symptoms occurred during the postoperative period**. Note that in 97.6% of the delirious cases (120 of 123), the initial symptom occurred within the first three days after surgery. POD, postoperative delirium.

One hundred twenty-three patients developed delirium after surgery, resulting in an overall postoperative delirium rate of 50.6% (123 of 243). Among patients who developed delirium, the median (interquartile range) time of occurrence of the initial delirious symptom was 1 (1 to 2) day after surgery. In 97.6% of the delirious cases (120 of 123), the initial symptom occurred within the first 3 days after surgery (Figure 2). The median duration of postoperative delirium (that is, the duration between the initial symptom and the last symptom) was 2 (1 to 4) days.

Four patients died within 28 days after surgery, resulting in an overall 28-day mortality rate of 1.6%. Two of them died of intractable ventricular fibrillation on the 1st and 3rd postoperative day, respectively. Because of unarousable sedation or coma, these two patients were not assessed for delirium. The other two patients died of circulatory failure and multiple organ failure on the 10th and 26th postoperative day, respectively. Both of them experienced early postoperative delirium.

**Figure 3:**
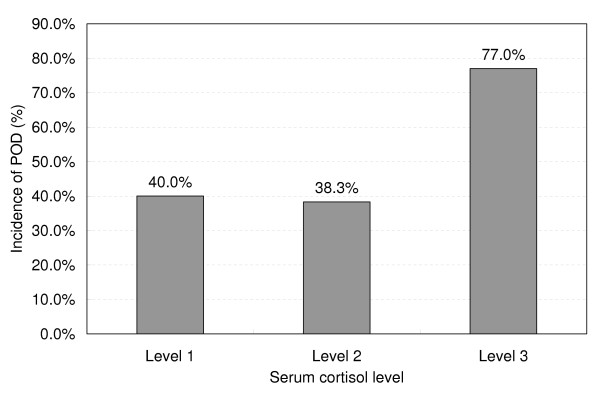
**Relationship between serum cortisol level and incidence of postoperative delirium (POD) after coronary artery bypass graft surgery**. Patients with a higher serum cortisol level had a significantly higher incidence of POD (*P *< 0.001). Level 1 indicates a serum cortisol concentration of less than 138 nmol/L, level 2 indicates a serum cortisol concentration of between 138 and 690 nmol/L, and level 3 indicates a serum cortisol concentration of greater than 690 nmol/L.

Variables that were significant in univariate analyses (*P *< 0.10) are listed in Table 4. Patients with high serum cortisol level had a significantly higher incidence of postoperative delirium (*P *< 0.001) (Figure 3). After the multicollinearity was tested, two variables were excluded from further multivariate logistic regression analysis because of high correlation with others (duration of anesthesia versus duration of surgery, Pearson correlation coefficient = 0.967, *P *< 0.001; serum cortisol concentration versus serum cortisol level, Spearman correlation coefficient = 0.867, *P *< 0.001). There was only a weak correlation between serum cortisol concentration and APACHE II (Acute Physiology and Chronic Health Evaluation II) score on arrival in the ICU (Kendall correlation coefficient = 0.122, *P *= 0.008). No significant correlation existed between serum cortisol concentration and duration of surgery (Pearson correlation coefficient = -0.003, *P *= 0.963), use of cardiopulmonary bypass (Kendall correlation coefficient = -0.018, *P *= 0.735), or duration of mechanical ventilation in the ICU (Pearson correlation coefficient = -0.018, *P *= 0.790). So the above four variables were included in the multivariate analysis. On the other hand, since most of the delirious cases were diagnosed on the first day after surgery, we included only the complications that occurred before or during the first postoperative day in the multivariate analysis.

**Table 4:** Predictors of postoperative delirium.

Variable	Univariate analyses^a^	Multivariate logistic regression analysis^b^	
		
	*P *value	Odds ratio (95% CI)	*P *value
Age, years	< 0.001	1.111 (1.065-1.159)	< 0.001
History of diabetes mellitus	0.035	1.905 (1.001-3.622)	0.049
Preoperative LVEF, percentage	0.026	-	-
Preoperative NYHA functional class	0.029	-	-
Preoperative EuroSCORE score	< 0.001	-	-
Duration of surgery, hours	0.013	1.360 (1.010-1.831)	0.043
Duration of anesthesia, hours^c^	0.020	-	-
Blood transfusion of at least 400 mL during surgery	0.048	-	-
Postoperative APACHE II score	< 0.001	-	-
Serum cortisol concentration, nmol/L^c^	< 0.001	-	-
Serum cortisol level, every level increase^d^	< 0.001	3.091 (1.763-5.418)	< 0.001
Postoperative LVEF, percentage	0.009	-	-
Postoperative cardiac insufficiency^c^	0.015	-	-
Postoperative arrhythmia^c^	0.065	-	-
Postoperative complications within 1 day^e^	0.001	2.485 (1.184-5.214)	0.016
Postoperative complications within 5 days^c,e^	< 0.001	-	-
Postoperative complications within 28 days^c,e^	< 0.001	-	-

^a^Occurrence of postoperative delirium was modeled as a function of a single predictor. ^b^Occurrence of postoperative delirium was modeled as a function of all predictors that differed (*P *< 0.10) in the univariate analyses. Excluded were nine cases with missing values for at least one of the risk factors in the model. Multivariate logistic regression analysis was performed by using a forward (conditional) stepwise procedure. ^c^Variable was not included in the multiple logistic regression analysis. ^d^The normal range is 138 to 690 nmol/L. Level 1 indicates a serum cortisol concentration of less than 138 nmol/L, level 2 between 138 and 690 nmol/L, and level 3 of greater than 690 nmol/L. ^e^Complications that occurred before or during the 1st, 5th, or 28th postoperative day. APACHE II, Acute Physiology and Chronic Health Evaluation II; CI, confidence interval; EuroSCORE, European System for Cardiac Operative Risk Evaluation; LVEF, left ventricular ejection fraction; NYHA, New York Heart Association.

As a result, 11 variables were consecutively subjected to a stepwise logistic regression analysis. Five were identified as independent predictors of postoperative delirium (Table 4). Of particular note was that high serum cortisol level was associated with significantly increased risk of postoperative delirium in this risk-adjusted analysis (odds ratio [OR] 3.091, 95% confidence interval [CI] 1.763 to 5.418; *P *< 0.001). Replacement of serum cortisol level with serum cortisol concentration in nanomoles per liter did not change the results, and high serum cortisol concentration remained an independent risk factor of postoperative delirium (OR 1.002, 95% CI 1.000 to 1.003; *P *= 0.006).

Comparison between patients with or without postoperative delirium showed that the former group had significantly more occurrences of postoperative complications, prolonged duration of ICU stay, and prolonged duration of postoperative hospital stay. The former group also tended to have greater medical expense during hospitalization (Tables 3 and 5).

**Table 5:** Outcomes of patients

Variable	Non-delirious patients (*n *= 120)	Delirious patients (*n *= 123)	*P *value
Number of postoperative complications per patient			0.001
0	92 (76.7%)	65 (52.8%)	
1	24 (20.0%)	51 (41.5%)	
2	4 (3.3%)	6 (4.9%)	
≥3	0 (0.0%)	1 (0.8%)	
Duration of ICU stay, hours	22.0 (21.0-46.0)	45.3 (22.8-87.3)	< 0.001
Duration of postoperative hospital stay, days	7 (7-7)	7 (7-10)	< 0.001
Total costs of hospitalization, ×1,000 CNY	54.9 (48.1-63.8)	57.6 (51.0-67.4)	0.057

Data are presented as number of patients (percentage) or median (interquartile range). CNY, Chinese Yuan; ICU, intensive care unit.

## Discussion

In the present study, we demonstrated that, in patients undergoing CABG surgery, elevated serum cortisol level on the first day after surgery was highly correlated with increased risk of postoperative delirium. Other independent risk factors included increasing age, history of diabetes mellitus, longer duration of surgery, and occurrence of complications within the first day after surgery. Our study also confirmed that outcomes were worse in patients who developed postoperative delirium: they had a higher incidence of postoperative complications, prolonged durations of postoperative ICU and hospital stay, and a tendency to greater medical expense during hospitalization.

The reported incidences of postoperative delirium varied from 3% to 72% after all types of cardiac surgery and from 3% to 50% after CABG surgery [2-6,28-32]. A recent study by Rudolph and colleagues [33] reported a rate of 52%. The reasons that produce this great variation include difference in patient population (such as age, severity of illness and type of procedure), sensitivity of the screening instrument, and local medical practice (such as routine practice and critical care environment). Although, in our study, patients were relatively young and underwent low-risk closed-chamber surgery [34,35], the incidence is higher than some previously reported ones. This is perhaps because we assessed delirium twice daily for five consecutive days after surgery and thus detected more delirious cases. Even though it was not found to be a significant risk factor, the frequent use of anticholinergics during surgery in our study is another possible reason for this higher incidence.

The cause of delirium is typically multifactorial [12]. Delirium occurs as a result of a complex interrelationship between predisposing and precipitating factors [12,36]. Numerous studies have been performed to find out the risk factors for developing postoperative delirium, and there is substantial heterogeneity in the findings [3-6,28-32]. Increasing age is a universally identified risk factor of delirium [2,12,36], suggesting that the naturally aged brain might be the basis of the occurrence of delirium. A history of diabetes mellitus is associated with increased incidence of almost all kinds of cerebral complications after cardiac surgery, including stroke [37], cognitive dysfunction [38], and delirium [39,40]. This is perhaps because long-standing diabetes mellitus increases the prevalence of intracerebral atherosclerotic disease [41]. On the other hand, a long duration of surgery indicates a more complex procedure and the occurrence of postoperative complications means a more eventful recovery, and both long duration of surgery and postoperative complications have also been found to be associated with the occurrence of delirium [3,42,43].

Preoperative psychiatric disorders, such as depression and cognitive impairment, are also strong predictors of postoperative delirium [33,44]. In the present study, only one patient who was previously diagnosed with schizophrenia and was taking antischizophrenic drugs at the time of surgery was excluded from the study for psychiatric reasons. This did not seem to produce patient selection bias.

It was reported that off-pump CABG surgery was associated with less frequent postoperative delirium [4]. However, this topic is controversial. Recent studies did not confirm that off-pump procedure or less cerebral emboli improved neurologic outcomes in patients undergoing CABG surgery [45,46]. Furthermore, it was found that systemic stress hormone response triggered by off-pump surgery was comparable with that after conventional on-pump surgery [47]. In the present study, there were no differences in the incidence of delirium (58/114 versus 65/129; *P *= 0.939) and the serum cortisol concentration (558.0 ± 342.6 nmol/L versus 542.9 ± 259.1 nmol/L; *P *= 0.702) between patients undergoing on- or off-pump surgery. We therefore combined on- and off-pump surgeries and included the type of surgery in the analyses. The results showed no significant relationship between the type of surgery and the occurrence of postoperative delirium.

It has long been known that stress and high circulating glucocorticoid level can produce deterioration in neuropsychological function [19]. Studies showed that persistently elevated glucocorticoid levels may affect neurochemical transmission and lead to structural changes in hippocampal neurons [48]. Psychiatric symptoms are common adverse effects in patients undergoing systemic corticosteroid therapy [20]. For patients after acute ischemic stroke, high serum cortisol level was significantly correlated to the presence of acute confusional state [49]. In the preliminary study by McIntosh and colleagues [50], the occurrence of postoperative delirium was associated with a significant and unusually prolonged increase in circulating cortisol level. In a recent study, Shi and colleagues [23] found that high serum cortisol level was associated with increased incidence of postoperative delirium after noncardiac surgery.

We did not monitor the time-course changes of serum cortisol level in our study. In the ISPOCD2 (International Study of Postoperative Cognitive Dysfunction), salivary cortisol concentrations were monitored for a 3-month period. Peak levels were found in the morning of the first postoperative day [51]. For patients undergoing cardiac surgery, studies also found that serum cortisol concentrations peaked in the first postoperative day or from 4 to 12 hours after surgery and then recovered gradually toward baseline during several days [52,53]. In this study, we collected blood samples in the early morning of the first postoperative day in order to get a relatively high serum cortisol level. The normal range of morning serum cortisol concentration in our hospital laboratory is 138 to 690 nmol/L, which is broadly the same as that of other laboratories [54]. For the convenience of analyses, we divided the serum cortisol concentrations into three levels according to the normal range (that is, level 1 is lower than 138 nmol/L, level 2 is within normal range, and level 3 is higher than 690 nmol/L).

Our study demonstrated, for the first time, that elevated serum cortisol level is significantly correlated with increased incidence of delirium in patients after cardiac surgery. In our results, the number of new delirious cases was highest on the first postoperative day and decreased rapidly across time, with 97.6% of the delirious cases occurring within the first three days after surgery. The median (interquartile range) duration of delirious symptoms was 2 (1 to 4) days. The time course of postoperative delirium is similar to the reported changes of serum cortisol level after cardiac surgery [52,53]. This accordance also indicates a relationship between serum cortisol level and occurrence of postoperative delirium.

It remains unknown whether hypercortisolemia is a cause or an effect of postoperative delirium. Studies found that an older population and patients with diabetes mellitus have an increased baseline cortisol level and an attenuated negative feedback mechanism that inhibits further secretion of cortisol and are more reactive to stressful stimuli [55-57]. In clinical settings, these two populations are prone to develop postoperative delirium [2-6,28-32,39,40]. An earlier study of delirium in patients with lower respiratory tract infection showed that older patients who were nonsuppressor on the dexamethasone suppression test were at increased risk for developing delirium during acute illness [58]. It is possible that abnormal hypothalamic-pituitary-adrenal function plays a basic role in the mechanism of delirium.

Surgery-related stress is not the only factor that contributes to the elevated cortisol level. In the present study, we did not find significant correlations between serum cortisol concentration and duration of surgery, use of cardiopulmonary bypass during surgery, or duration of mechanical ventilation in the ICU. Velissaris and colleagues [47] reported a similar cortisol response curve in patients undergoing on- and off-pump surgery. Other conditions (such as anxiety, depression, and cognitive impairment) are also related to higher serum cortisol concentration [55,59]. However, we did not perform screen tests for these conditions in this study. On the other hand, 20 patients (8.2%) in our study had a serum cortisol concentration that was lower than normal in the morning of the first postoperative day. The possible reason is that etomidate was used for anesthesia induction in all patients. It has been found that a single dose of etomidate can cause adrenal inhibition for 12 to 24 hours [60]. However, this did not seem to produce significant adverse effects in our study since only one of these patients needed inotropic therapy for more than 24 hours and no glucocorticoid replacement therapy was administered in the ICU.

There are several limitations of this study. First, we did not perform baseline psychiatric and cognitive screening tests. It was reported that preoperative mental disorders (such as depression, cognitive impairment, and dementia) are strong predictors of postoperative delirium [33,44]. These factors are not included in the multivariate analysis in our study and thus may interfere with the final results. Second, serum cortisol concentrations were not measured at baseline. They were measured at only one time point after surgery. We were unable to determine whether patients with elevated baseline serum cortisol level were more prone to develop postoperative delirium and whether the time course of postoperative delirium was exactly correlated with that of serum cortisol level. Third, we did not observe the long-term effects of surgery on postoperative cognitive dysfunction. Therefore, we could not, as Koster and colleagues [61] had, determine whether there is an association between postoperative delirium and long-term outcomes. Fourth, our study did not reveal the causal relationship between the elevated cortisol level and the occurrence of delirium. Hypercortisolemia may have a direct impact on delirious symptoms, but it is also possible that hypercortisolemia merely reflects the stress associated with delirium [62]. Therefore, further study is needed to illuminate the mechanisms by which circulating cortisol level may affect delirium.

## Conclusions

The results of our study showed that delirium was a common complication after CABG surgery. High serum cortisol level was associated with increased risk of postoperative delirium. Patients who developed delirium had outcomes that were worse than those who did not.

## Key messages

• Postoperative delirium was a common complication after coronary artery bypass graft surgery.

• High serum cortisol level was associated with increased risk of postoperative delirium. Whether this relationship is causal or due to other confounders is still unclear.

• Patients who developed delirium had outcomes that were worse than those who did not.

## Abbreviations

CABG: coronary artery bypass graft; CAM-ICU: Confusion Assessment Method for the Intensive Care Unit; CI: confidence interval; DSM-IV: *Diagnostic and Statistical Manual of Mental Disorders, Fourth Edition*; ICU: intensive care unit; OR: odds ratio; RASS: Richmond Agitation Sedation Scale.

## Competing interests

The authors declare that they have no competing interests.

## Authors' contributions

D-LM assessed the occurrence of postoperative delirium, collected the patients' data, and drafted the manuscript. D-XW designed the study, performed the statistical analysis, and revised the manuscript and approved the final version to be published. L-HL, Q-JY, and C-XS participated in the study design and collected the patients' data. G-JS and JL performed the measurements and collected the patients' data. All authors read and approved the final manuscript.
